# Cataloguing Treatments Discussed and Used in Online Autism Communities

**DOI:** 10.1145/3038912.3052661

**Published:** 2017-04

**Authors:** Shaodian Zhang, Tian Kang, Lin Qiu, Weinan Zhang, Yong Yu, Noémie Elhadad

**Affiliations:** † Department of Biomedical Informatics, Columbia University, New York, NY, US; ‡ Apex Data and Knowledge Management Lab, Shanghai Jiao Tong University, Shanghai, China

**Keywords:** Natural Language Processing, Autism, Online Health Community, Treatment, Conditional Random Fields

## Abstract

A large number of patients discuss treatments in online health communities (OHCs). One research question of interest to health researchers is whether treatments being discussed in OHCs are eventually used by community members in their real lives. In this paper, we rely on machine learning methods to automatically identify attributions of mentions of treatments from an online autism community. The context of our work is online autism communities, where parents exchange support for the care of their children with autism spectrum disorder. Our methods are able to distinguish discussions of treatments that are associated with patients, caregivers, and others, as well as identify whether a treatment is actually taken. We investigate treatments that are not just discussed but also used by patients according to two types of content analysis, cross-sectional and longitudinal. The treatments identified through our content analysis help create a catalogue of real-world treatments. This study results lay the foundation for future research to compare real-world drug usage with established clinical guidelines.

## 1. INTRODUCTION

Online health communities (OHCs) provide means of exchanging information and social support for a vast amount of patients, especially individuals with chronic or life-threatening diseases. A wide range of studies have been carried out for purposes such as creating social support interventions [28, 19, 31], understanding patient behaviors [18, 36], assisting community facilitators [24], and mining critical disease- or medication-specific information [30, 35, 38]. Information related to disease treatment, such as medications, therapeutic protocols, and surgeries, have been particularly prevalent in online health discussion. One specific research question has remained unexplored thus far is which treatments are actually taken or adopted by online health community members in their real lives, as opposed to merely discussed within a community. This question is of interest to patients, clinical practitioners, and health researchers alike: patients want to know about the drugs and protocols their peers rely on, as means to inform their own decision making. Clinicians and health researchers can learn about adherance to various treatments. For health researchers, public online health communities provide massive cohorts of patients who are potential subjects of post-market research. For example, researchers may be interested in what treatments are actually consumed by patients, in contrast to what are suggested by established clinical guidelines. Traditionally, it is usually difficult to carry out such research analysis outside of clinical setting, which provides unprecedented opportunities to rely on content analysis of OHCs and social media. For example, to compare clinicians’ and patients’ perspectives on the symptomatic treatment of ALS, Nakamura et al. conducted a study by comparing data from a traditional survey study of clinicians with data from an OHC, PatientsLikeMe [27].

In most health communities, patients discuss and report on their treatments through posts, with free-text narratives. Critical to our task at hand is thus a method that can automatically identify signals of actual treatment usage from OHC content, especially OHC text. The task is not as trivial as simply extracting mentions of treatment from user posts. One particular challenge of connecting online content with reality should be tackled first: messages conveyed through content may not necessarily indicate the happening of corresponding events in real lives. In the case of treatment-related discussions, discussing a treatment online does not always indicate the action of adopting the treatment. Particularly, many of the mentions of treatments may not be attributed to the patients themselves. For example, users in OHCs may discuss related scientific findings about a treatment, in which a large number of treatment names may occur. Such mentions do not indicate any actual usage of the drugs, and therefore should be excluded when cataloguing patients’ treatment usage.

The context for our study is Autism Spectrum Disorder (ASD), a range of developmental disorders originating in early childhood, and two ASD online communities. Unlike other disease-specific communities where participants are primarily patients or survivors, autism communities’ members are mostly parents of children on the spectrum. In ASD forums, members primarily discuss their children’s diagnoses and treatments and a wide range of topics related to their children’s lives. They also rely on the community to exchange information and support about themselves as caregivers. As such, in order to build up catalogues of treatment for patients of interest (i.e., children with ASD), it is important to distinguish between treatments attributed to patients themselves and to the caregivers.

In this paper, we aim to build a catalogue of treatments for children with ASD through text mining of OHCs. Our approach consists of two steps: extracting mentions of treatment in OHC posts and classifying treatment attributions, i.e., attributing the treatment mention to a patient, a caregiver, someone else, or as a generic mention. We show that treatment mentions and their attributions could be identified jointly through an end-to-end classifier based on conditional random fields (CRF). Results of this study can help investigate further real-world use of ASD treatments as derived from caregivers’ discussions: the catalogue of treatments, and even their established prevalence of use in a community, can be compared with established clinical guidelines, and help study the gap between clinical expectation and patients’ daily lives. In this paper, we will also take temporal information into consideration, investigating how members’ perceptions and usages of treatments change throughout their community participation, thus investigating whether there is any influence from the community in the decision making process of community members when it comes to treatments.

## 2. BACKGROUND

### 2.1 Autism Spectrum Disorder (ASD)

Autism Spectrum Disorder (ASD or autism) is a neurodevelopment disorder characterized by deficits in social communication, social interaction, as well as the presence of restricted, repetitive behaviors [1]. Autism statistics from the U.S. Centers for Disease Control and Prevention (CDC) identify around 1 in 68 American children as on the autism spectrum, a ten-fold increase in prevalence in 40 years. ASD affects over 3 million individuals in the U.S. and tens of millions worldwide [6].

ASD is typically diagnosed on the basis of behavioral symptoms, without reference to etiology. However, considerable research has been devoted to investigations of etiological factors. To date, there is no accepted single cause of ASD, although there are numerous theories and studies suggest that autism results from different sets of causal factors, such as genetic, neurobiological, and environmental, that manifest in characteristic behavioral symptoms [5, 15]. The most obvious signs of autism and symptoms of autism tend to emerge between 2 and 3 years of age. Currently, for diagnosis of ASD, no established medical test was recognized, as the symptoms of autism vary [7]. Instead, specially trained physicians and psychologists administer autism-specific behavioral evaluations.

The treatment or intervention of ASD can involve behavioral treatments, medicines or both. Given the nature of autism and the needs of individuals with ASD, family involvement serves as a key role during the treatment [26]. Most comprehensive programs for individuals with ASD offer parents/caregiver training.

In fact, beyond the impact of ASD on the patients themselves, ASD impacts families as a whole. Caregivers as such have a with a wide range of emotional, instrumental, and informational needs. Online autism communities are a place for exchange of support for these three types of needs. Maybe because the etiology and mechanisms of ASD are still enigmatic, ASD communities contain much discussions and opinions amongst caregivers about causes and efficacy of various treatments, including misinformation (e.g., vaccines as a cause to autism) [11]. There is a critical need to understand to which extent caregivers’ understanding of ASD and treatment decisions align with current clinical characterization of the disease.

### 2.2 Named Entity Recognition and Attribution Identification

In this paper, our primary task is to recognize treatments mentioned in OHCs and then distinguish their attributions. The first step, recognizing treatment names, is traditionally in the realm of named entity recognition (NER). One of the most important use case of clinical NER is mining patient related healthcare knowledge from Electronic Health Records (EHR), which contain abundant information in free-text format. NER is one of the fundamental issues in information extraction of EHR [23]. Both supervised and unsupervised approaches are deployed in clinical NER systems. One of the most mature systems for clinical text processing is MedLEE [14], which uses heuristic rules and patterns for identifying clinical problems, treatment and events. Zhang et al. developed an unsupervised clinical NER system by generating seed terms from Unified Medical Language System (UMLS) [39]. Learning-based methods have been rapidly advanced in the past 5 years [16]. Representative ones include conditional random fields (CRF) and structured support vector machines (SVM) [34]. For example, the best system reported in the i2b2 NER challenge from deBrujin et al. used Semi-Markov (F score 85.2%) [10], followed by the system by Jiang et al. using CRF (F-score 83.9%) [20].)Mining important clinical entities and events in social media data for health related purpose is also a promising field, especially from the perspective of public health. Social networks have seen an unprecedented growth worldwide. A large population of patients are actively involved in sharing and posting health related information in social networks [32].

Pertaining to the second task of this paper, attribution identification, a recent survey [13] has revealed that 34% of caregivers and 20% of patients read or watch other’s commentary or experience online. In addition, 11% of caregivers and 6% of patients share experience or post questions online. Particularly, in health related social platforms, users share and discuss their health-related experience with others facing similar problem, including use of drugs, side effects and treatments, which makes such social networks unique and robust source of information such as drugs and treatments [32]. Numerous studies have been published recently in this field, including studies on pharmacovigilance [17], identifying smoking cessation patterns [22], identifying user social circles with common experiences such as drug abuse [4], and tracking infectious disease spread [12, 2, 29].

## 3. METHODS

### 3.1 Dataset

The dataset of autism forums used in this paper were collected from two publicly available sources: autismweb.com and autism-pdd.net, which are primarily for ASD patients and caregivers. The forum from autism-pdd.net was officially closed in 2015 and could no longer be accessed. We crawled all content that was public available from these two forums in March 2015. These two forums are designed for the same audience and thus have similar functionalities, but the forum from autismweb.com is significantly larger than the one from autism-pdd.net. As such, we merged these two forums into one single dataset, with following information available: sub-forums, threads, posts, and authors. Detailed descriptive statistics of this dataset can be found in Table 1.

### 3.2 Annotation

Five types of attributions were considered in the manual annotation, with descriptions given in Table 2. In general, the labels were designed to reflect whom the treatment is tied to. We consider four types of attributions: mentions attributed to the patients (ASD children), the caregivers (usually parents of children, who actually participated in the discussions), others (either other people in the community or other people in the real world), and no one (i.e., general discussions). Moreover, as we discussed previously, an entity of a treatment attributed to a patient does not necessarily indicate an actual history of usage. For instance, in “The doctor suggested to put my son on risperdal”, although the mention “risperdal” is associated with the patient (my son), it is not clear whether the drug is eventually prescribed by the doctor and hence taken by the patient. Therefore, in order to support subsequent user modeling in which we establish a treatment catalogue for each user, in the annotation schema we further distinguish mentions of treatments attributed to patients which do and do not indicate actual usage or usage history, i.e. *pt* and *pt-gen* in Table 2.

A randomly sampled 500 posts were extracted and split into two sub-sets, with 50 posts overlapping (i.e. first set from post 1 to post 275, second set from post 225 to post 500). Two annotators were asked to 1) identify mentions of treatments (entities) from text, and 2) annotate the attribution label according to Table 2 for each mention. It is noteworthy, however, that the annotators were asked to classify attributions locally, without considering context which may shift the attribution of a mention. For example, in “The doctor suggested to put my son on risperdal.....My son tried risperdal and...”, the first mention of risperdal should be labeled as *pt-gen*, even if following context indicates an actual usage of the same drug. In the annotation for this task, we did not consider co-references, e.g., pronouns which refer to treatments. Since our purpose is to find treatment history of patients, ignoring co-references should not affect the final results significantly as long as all treatment names are properly identified.

The annotation started with each annotator coding the overlapping part of the two sets, on which we tracked inter-rater agreement. Our annotators reached a Kappa of 0.77 after 3 rounds of pre-annotation and disagreement resolving. The remaining parts of the two sets were coded by the two annotators independently. In total, 4,264 mentions of treatments were identified from the 500 posts. Among them, 434 were annotated as *pt-gen*, 1,830 as *pt*, 210 as *others*, 95 as *cg*, and 1,635 as *gen*.

### 3.3 Models and Experiments

We base on the popular sequence labeler, the conditional random fields (CRF), to jointly identify treatment mentions and their attributions. Three separate sets of evaluations were carried out for this task. Since the CRF model handles term identification and term classification jointly, it is necessary to evaluate these two separate steps in a explicit way. As such, the first set of evaluations is to examine how well the classifier can detect treatment entities, regardless of their attribution labels. The second set of evaluations takes attributions into consideration and evaluates the end-to-end performance of the method on the task. Finally, in our particular scenario of application in which we aim at building up treatment catalogue for each patient, we are more interested in one attribution label, the *pt* class. Therefore, one additional evaluation is also carried out in which only two attribution labels are considered, *pt* and *non-pt*. The *non-pt* class is simply the aggregation of all attribution labels other than *pt*. For each set of evaluations, we report the performance of CRF model with different sets of features, ranging from basic lexical ones to syntactic features and information from context posts. We also compare the system with baselines. The baseline system for mention identification is based on a dictionary match using Consumer Health Vocabulary [33]. The baseline system for attribution classification is a logistic regression classifier with lasso regularization using the same set of features.

Features we used in this study are described as follows: *Lexical features* refer to words, lemmas, part-of-speech tags of the content. In this study, we rely on the existing open source tools, the OpenNLP [3], to extract these features from raw content. The feature also includes occurrences of non-semantic tokens, such as question marks, exclamation marks, and mentions of user names.

*Syntactical features* are ones relying on the parse tree of the sentences. In particular, subject (usually an NP) and predicate (usually a VP) of sentences, as well as the lengths of paths from current token to subject and predicate, are used as features. Sentences were parsed by the StanfordNLP toolkit [21] in our experiments.

*Semantic features* refer to those ones representing domain knowledge, relying on dictionary matching according to following lexicons: WordNet [25], UMLS metathesaurus [8], and Consumer health vocabulary [33].

## 4. RESULTS

### 4.1 Treatment Mention Identification

Table 3 lists performance measured by precision, recall, and F score for the treatment name identification, regardless of attribution types. All CRF-based systems, no matter what the features are, outperform the baseline (dictionary matching based on Consumer health vocabulary) significantly. However, syntactical features do not help the system performance. It seems to suggest that regardless of the treatment attributions, lexical features alone (including ones based on lexicons) are sufficient to identify the treatment mentions for CRF model.

### 4.2 End-to-end Evaluation

Performance of the end-to-end evaluation of joint treatment mention detection and attribution classification is given in Table 4. A true positive in this evaluation is a recognized treatment mention with both boundary and attribution correctly identified. As a result, it is a more challenging task since either an incomplete boundary or an incorrect attribution label will make the prediction counted as an error. The overall micro averaged F score is around 50 to 60, which varies by different feature sets. Syntactic features, which represent more global information from the whole sentence, are decisive in this task. Compared with the standalone evaluation of treatment identification, it seems to suggest that syntactic features are helpful for attribution classification, but not entity recognition.

The baseline systems use dictionary matching for term identification, and logistic regression with corresponding features for attribution classification. All baseline systems underperform their CRF counterparts significantly. However, the performance of baseline systems may just be compromised by the weak dictionary matching baseline for term identification. In order to compare CRF and logistic regression more fairly in attribution classification. We also carried out a set of additional experiments, where CRF’s results on term identification were given to logistic regression as input for attribution classification. The results are given as basline+ in Table 4. It can be seen that systems based on CRF still outperform logistic regression, which suggests that attribution identification benefit from sequence learning.

Across the five attribution categories, our method is able to classify *gen* and *pt* better than the other three. This is primarily because of the distributions of these attributions in the training and test datasets - *pt* and *gen* are the most dominant attributions. Fortunately, in our downstream applications, building up treatment catalogues for patients, only treatment mentions with *pt* attribution will be used. To see if excluding other attributions from the dataset to make the classification as a binary choice (*pt* vs. *non-pt*) can help boost the accuracy of identifying mentions attributed to patients, we carried out an additional evaluation in which *gen*, *others*, *pt-gen*, and *cg* were merged into one class. The performance is given in Table 5. Compared with Table 4, accuracy of identifying Patient is boosted for around 4–5 percent, although the dataset, feature, and model keep exactly the same ones. The results suggest that properly formulating the task and setting up the target categories make significant difference in this type of tasks.

### 4.3 Creating Treatment Catalogues for Members

We applied the best system to the entire un-annotated dataset. In total, 164,335 mentions of 3,981 different treatment terms were identified in the entire ASD dataset. In average, around every three posts have one treatment mention. *pt*, which represents that a mention is attributed to patients of interest, is the most dominant attribution label, with 79,778 mentions of 3,552 treatment terms identified. Since some of the terms may refer to the same treatment (e.g. chelation and chelating), actual number of treatment identified may be less. 71,1102 mentions of 3,622 treatment terms, 7,783 mentions of 1,142 treatment terms, 5,297 mentions of 915 treatment terms, and 275 mentions of 176 treatment terms are identified for attribution *gen*, *pt-gen*, *others*, and *cg*, respectively.

The original top ten most frequent treatment terms with corresponding numbers of mentions for each attribution class are given in Table 6. The lists contain common treatment options for ASD patients, as well as alternative therapies. Prevalence of the same treatment in different attribution classes may differ. For instance, although chelation is the most prevalent treatment discussed in the forum, and is particularly popular when attributed to general discussions (See the class *gen* in Table 6 ), number of mentions of chelation which attributed to the users’ actual usage is not that dominant. It is interesting that alternative therapies, such as probiotics and vitamins, are used by patients in the forum almost as frequently as conventional drugs such as Risperdal. Moreover, it is surprising that almost all the top ten terms identified for each attribution class are indeed either treatment options or nutritional supplements, with only one false positive appeared in the list of Caregiver (cab). Given the broad coverage of treatments identified, the high precision of the top term lists indicate a successful application of the our method to identify treatment terms. However, some of the terms identified in specific attribution classes are questionable. In particular, treatments attributed to caregivers in current result are mostly treatment options for ASD, which are likely to be caused by incorrect classifications.

**Figure 1:**
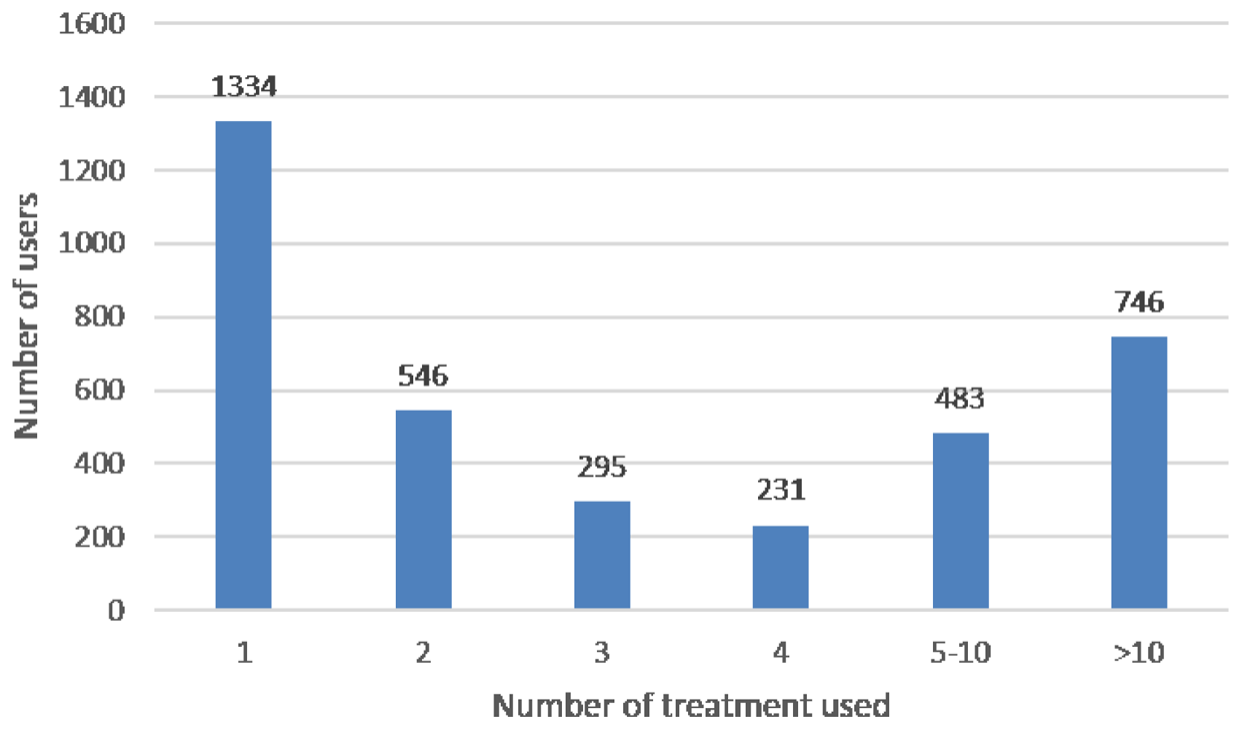
Distributions of number of users, by number of used treatment. The x axis is the number of used treatment identified, and the y axis is the number of users.

After identifying attributions of treatment mentions, for each user we are able to create a treatment catalogue in which all treatments attributed to their ASD children are recorded. We obtain this by simply aggregating all treatment mentions whose attribution are *pt*, in all the posts of individual users in the forum. As such, we are able to create treatment catalogues for 3,635 members. Among them, 2,301 have tried more than one treatment according to the identification. Distributions of number of users, by number of used treatment, is given in Figure 1. Most of the members have tried multiple treatments, which is consistent with the fact that parents tried various treatments as well as supplements for their ASD children, since autism is complex and hardly be curable with standard conventional protocols.

Table 7 shows the top ten treatments most used by members in the autism communities. The difference between this table and the *pt* columns in Table 6 is that multiple mentions of a treatment of the same attribution posted by one user will only be counted once in this table. As such, numbers in Table 7 represents the true prevalence of treatment usage among autism forum users, rather than frequencies of mentions. Chelation, as a controversial therapy which lacks sufficient scientific evidence of effectiveness, attracts a lot of discussions in the forums, according to its overall frequency in Table 6. However, it only ranks 3rd as the mostly used treatment by patients. On the contrary, probiotics as a nutritional supplement, and speech therapy as a well-established psychosocial therapy for ASD children, are more popular in real practice.

### 4.4 Longitudinal analysis of treatment catalogues of members

**Figure 2:**
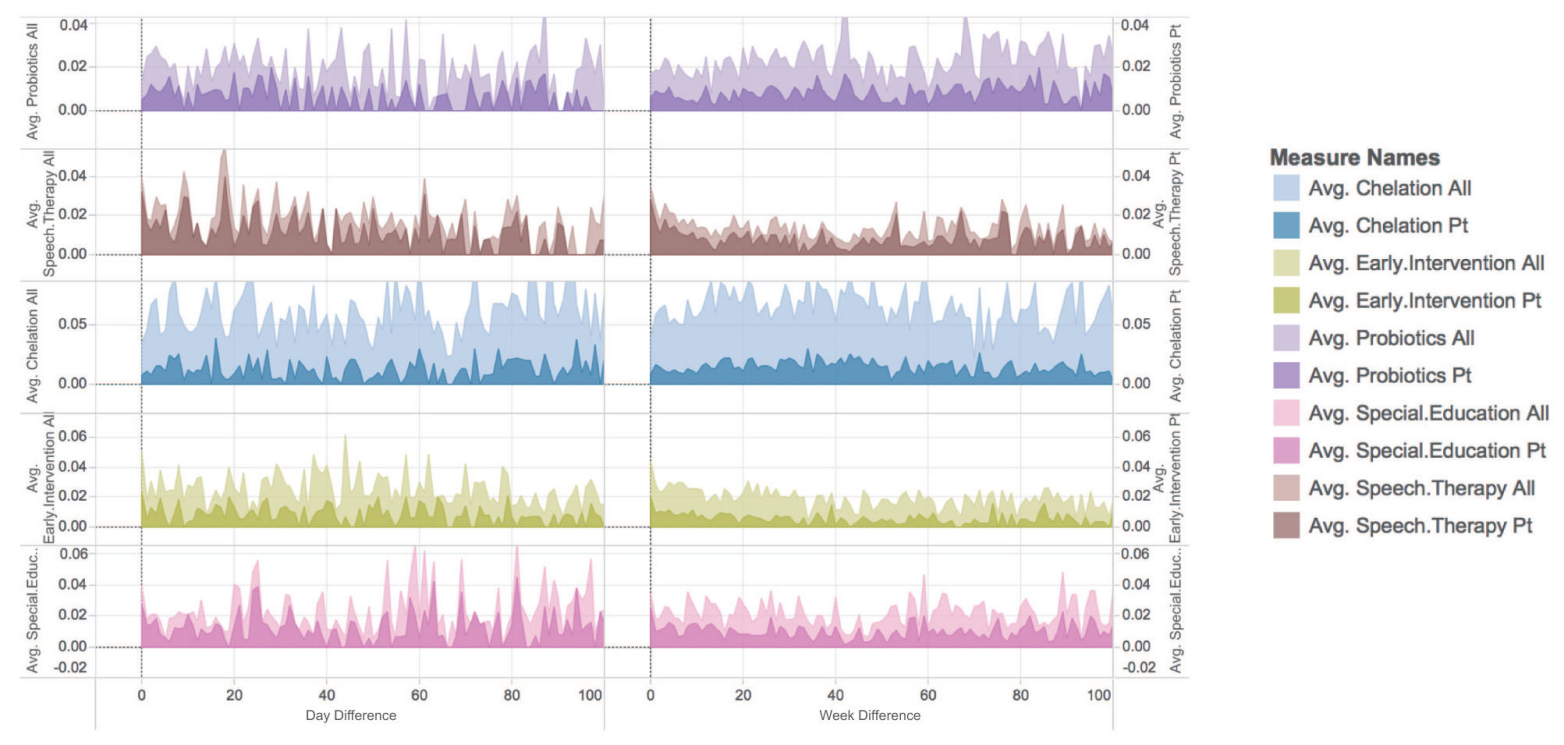
Changes of frequencies (mention per post) of top five treatments in autism communities, since members joining the community. Two separate X-axes represent views in weeks (right) and in days (left), respectively. Variables (measure names) ending with “all” represent total frequencies of mentions of corresponding treatment, regardless of their attribution types. Variables ending with “pt” represent frequencies of mentions of attribution type *pt*.

We also investigate how frequencies of treatment mentions change through time, and how the patterns differ across attribution types. Specifically, treatment mentions of attribution type *pt* and ones of other types are considered separately. We illustrate how frequencies of mentions change through time in weeks and in days since members joining the community in Figure 2.

In general, no clear pattern can be identified for each treatment. Members do not necessarily focus on certain treatment at the beginning stage of participation. In the long run, members keep discussing treatment options throughout their participation, with no decline in frequencies of mentions of any terms significantly.

In terms of frequencies of mentions attributed to patients, it was expected that such mentions should occur more frequently at the initial stage of participation, when members join the community and introduce conditions and current treatment adoptions of their ASD children. However, such pattern is not found in our analysis. On the contrary, frequencies of mentions attributed to patients fluctuate with total frequencies, and maintain substantial percentages throughout members’ participation. One possible explanation is that members try different treatment options for their children at different times, and keep updating about their effectiveness in the forums.

## 5. DISCUSSION AND CONCLUSION

The results suggest that abundant treatment options, ranging from conventional therapies to alternative ones, are discussed in the autism forums. Mentions of treatments are attributed to different stakeholders of autism care such as patients and caregivers. In the autism forums, most of the treatment discussions are attributed to ASD children of community members. Although not all mentions of treatment indicate actual adoption, around 90% of treatment mentions attributed to patients (ASD children) represent an ongoing treatment or a history of usage. Specifically, members keep updating status of their kids as they are treated, in which massive amount of treatment mentions occur. Members of the autism forums also discuss therapies frequently on issues like scientific evidence of effectiveness and information received from health professionals and online sources. A small proportion of treatment mentions are attributed to the caregivers themselves as well as other people in the community or in their real lives.

Our study also demonstrates that it is possible to rely on automated content analysis to overcome the issue by identifying attributions of extracted information and further filtering out information that is not associated with real actions. Similar issues also exist in studying other characteristics of OHC members (e.g. connecting sentiment expression to actual emotion), and should be the focus of future work.

We notice that some of the treatments, such as chelation, are discussed prevalently in the communities. However, they are not necessarily options that are mostly taken in practice. Within the top treatment list that represents actual usage, non-chemical psychosocial interventions such as speech therapy and special education are popular, although they are not necessarily the most popular ones under discussion. Our results provide a clear evidence that users’ perceptions, and hence actual adoptions, of treatment may not be accurately reflected by popularities in discussions, not to mention merely frequencies of certain keywords. More broadly, the results remind us that when connecting online content to members’ real life actions in a quantitative way, hidden information (e.g. attributions) of content must be taken into account to avoid mis-interpretation of results.

Longitudinally, we found that members discuss treatment therapies with quite constant frequency in the communities throughout their participation. No clear pattern could be identified in terms of how sustained participation affects frequencies of discussions of certain treatments. The finding is different from previous research findings where user sentiment and topics both show clear patterns longitudinally [40, 37]. Moreover, frequencies of mentions attributed to patients fluctuate with total frequencies, and maintain substantial percentages in all the mentions throughout members’ participation, which is somewhat counter-intuitive. In the future work, it is therefore an interesting question to explore how and why members keep mentioning treatment attributed to their ASD children throughout their participation.

The most important building block of future work following this study is to compare the list of treatments discovered in OHCs automatically by the computational tool with established clinical guidelines. For example, while effectiveness of chelation is still under investigation by researchers [9], it already becomes a rather popular choice among autism community members. It is therefore critical to further quantify how broad the gap is between established guideline and patients’ actual practice. The future work will contribute to understanding how information support and consumption in OHC affect members’ decision makings regarding disease management, and hence how OHC participation makes physical and psychological impact.

## Figures and Tables

**Table 1:** Descriptive statistics of the ASD dataset

Number of sub-forums	16
Number of threads	61,817
Number of posts	551,029
Number of authors	10,210

**Table 2:** Attribution labels for treatment mentions, their descriptions and their counts in dataset.

Attribution label	Description	Count
*pt*	Mention of treatment which indicates an actual usage or usage history of the patients of interest, usually children of the users in this particular study.	1830
pt-*gen*	Mention of treatment tied to the patient but does not indicate actual usage.	434
*cg*	Mention of treatment tied to the caregiver of the patient, usually the user herself.	95
*others*	Mention of treatment tied to specific individuals other than the caregiver or the patient. Can be other members in the community, or other people in the author’s real life.	210
*gen*	Mention not tied to a specific individual.	1635

**Table 3:** System performance for binary treatment mention detection with different types of features.

Features	Precision	Recall	F
Baseline	78.2	56.5	65.6
lexical	82.1	83.6	82.9
lexical+semantic	81.4	83.8	82.6
lexical+semantic+syntactical	81.0	83.5	82.2

**Table 4:** System performance (F score) for joint treatment detection and attribution classification with different types of features. The baseline systems use **dictionary matching** for term identificaiton, and logistic regression with corresponding features for attribution classification. The baseline+ systems use **CRF** for term identificaiton, and logistic regression with corresponding features for attribution classification.

Features	micro	cg	gen	others	pt	pt-gen

baseline (lexical)	33.4	9.8	31.2	29.1	45.0	13.4
baseline+ (lexical)	52.7	17.6	54.4	34.8	60.8	15.6
crf (lexical)	55.4	18.2	56.0	37.0	61.6	19.2

baseline (lexical+semantic)	34.1	10.2	31.1	29.0	45.6	14.5
baseline+ (lexical+semantic)	52.9	17.5	55.1	35.4	60.9	17.0
crf(lexical+semantic)	56.1	18.1	57.4	36.8	61.7	20.9

baseline (lexical+semantic+syntax)	35.0	14.6	29.9	31.2	44.1	15.4
baseline+ (lexical+semantic+syntax)	60.3	17.9	62.8	45.5	62.9	30.0
crf (lexical+semantic+syntax)	62.3	18.2	64.1	51.6	66.8	34.1

**Table 5:** System performance (CRF) for mentions with *pt* attribution with different types of features, when all other types of attributions are merged into one as *non-pt*.

Features	Prec. (pt)	Rec. (pt)	F (pt)

lexical	61.0	56.6	58.7
lexical+semantic	63.0	58.9	60.9
lexical+semantic+syntax	68.7	64.8	66.7

**Table 6:** Top 10 treatment with number of mentions for the five attribution classes, identified in the entire data set.

Term	Frequency	Term	Frequency
*pt*		*pt-gen*	

chelation	4935	chelation	1259
probiotics	2498	probiotics	389
zinc	2011	chelating	210
enzymes	1705	speech therapy	99
melatonin	1425	probiotic	98
special education	1287	activated charcoal	77
antibiotics	1283	nystatin	75
speech therapy	1245	melatonin	73
early intervention	1061	calcium	70
magnesium	889	early intervention	66

*cg*		*others*	

chelation	16	probiotics	424
progesterone	7	chelation	408
probiotics	7	probiotic	163
cod liver oil	5	chelating	150
chelator	4	melatonin	121
cab	4	enzymes	117
molybdenum glycinate chelate	4	zinc	114
sensory integration	4	risperdal	80
aloe vera	3	charcoal	77
pyridoxine hydrochloride	3	homeopathy	76

*gen*			

chelation	8341		
vitamin	1418		
early intervention	1268		
probiotics	1267		
special education	1153		
chelator	910		
vitamins	886		
melatonin	877		
homeopathy	862		
thimerosal	801		

**Table 7:** Top 10 treatment by number of users, identified in the ASD data set.

Term	Number of users

probiotics	819
speech therapy	565
chelation	520
early intervention	475
special education	395
melatonin	391
antibiotics	381
enzymes	352
zinc	332
vitamins	283
